# Comparing the Effect of Naturally Restored Forest and Grassland on Carbon Sequestration and Its Vertical Distribution in the Chinese Loess Plateau

**DOI:** 10.1371/journal.pone.0040123

**Published:** 2012-07-02

**Authors:** Jie Wei, Jimin Cheng, Weijun Li, Weiguo Liu

**Affiliations:** 1 State Key Laboratory of Loess and Quaternary, Institute of Earth Environment, Chinese Academy of Sciences, Xi'an, Shaanxi, China; 2 Graduate School of Chinese Academy of Sciences, Beijing, China; 3 State Key Laboratory of Soil Erosion and Dryland Farming on the Loess Plateau, Institute of Soil and Water Conservation, Chinese Academy of Sciences and Ministry of Water Resources, Yangling, Shaanxi, China; 4 Administrative Office of Yunwu Mountain, Guyuan, Ningxia, China; 5 School of Human Settlement and Civil Engineering, Xi'an Jiaotong University, Xi'an, Shaanxi, China; DOE Pacific Northwest National Laboratory, United States of America

## Abstract

Vegetation restoration has been conducted in the Chinese Loess Plateau (CLP) since the 1950s, and large areas of farmland have been converted to forest and grassland, which largely results in SOC change. However, there has been little comparative research on SOC sequestration and distribution between secondary forest and restored grassland. Therefore, we selected typical secondary forest (SF-1 and SF-2) and restored grassland (RG-1 and RG-2) sites and determined the SOC storage. Moreover, to illustrate the factors resulting in possible variance in SOC sequestration, we measured the soil δ^13^C value. The average SOC content was 6.8, 9.9, 17.9 and 20.4 g kg^−1^ at sites SF-1, SF-2, RG-1 and RG-2, respectively. Compared with 0–100 cm depth, the percentage of SOC content in the top 20 cm was 55.1%, 55.3%, 23.1%, and 30.6% at sites SF-1, SF-2, RG-1 and RG-2, suggesting a higher SOC content in shallow layers in secondary forest and in deeper layers in restored grassland. The variation of soil δ^13^C values with depth in this study might be attributed to the mixing of new and old carbon and kinetic fractionation during the decomposition of SOM by microbes, whereas the impact of the Suess effect (the decline of ^13^C atmospheric CO_2_ values with the burning of fossil fuel since the Industrial Revolution) was minimal. The soil δ^13^C value increased sharply in the top 20 cm, which then increased slightly in deeper layers in secondary forest, indicating a main carbon source of surface litter. However the soil δ^13^C values exhibited slow increases in the whole profile in the restored grasslands, suggesting that the contribution of roots to soil carbon in deeper layers played an important role. We suggest that naturally restored grassland would be a more effective vegetation type for SOC sequestration due to higher carbon input from roots in the CLP.

## Introduction

The biogeochemical cycles of carbon in terrestrial ecosystems have received worldwide attention in recent years due to the increased interest in greenhouse gas emissions and global warming. Soil organic carbon (SOC) holds 3.3 times as much carbon as the atmospheric pool and 4.5 times as much carbon as the biotic pool and is a major part of the terrestrial carbon reservoir [1]. Considerable research has reported that land use change may lead to carbon release or sequestration [2]–[3] and ultimately influence the concentration of CO_2_ in the atmosphere [4]–[5].

The Chinese Loess Plateau (CLP) is an important geological feature that has an important influence on the global carbon cycle [6]. The amount of vegetation coverage is relatively low in the CLP due to its vulnerable ecological environment. Since the 1950s, the Chinese Government has made great efforts to control soil erosion and restore ecosystems [7]. In 1999, the national “Grain for Green” project was launched in Northwest China by the Chinese government for ecological rehabilitation and long-term vegetation restoration, leading to faster conversion from sloping farmland to forest and grassland [8]. Previous research indicates that at least part of the SOC, depleted by cultivation, can be recovered if the native ecosystem is reestablished [9]–[10]. Therefore, assessing the potential of soil carbon storage is important for accurately evaluating the soil carbon pool in the natural vegetation restoration regions in the CLP.

The regional distribution of vegetation restoration types leads to different effects on SOC sequestration as a result of the variation in precipitation/soil moisture [11], terrain [12] or bedrock [13]. The subject investigated in this research is secondary forest and restored grassland because they are the most common types of vegetation restoration in the CLP. Previous studies conducted in this region have quantified ecosystem services changes under policy-driven large scale ecological restoration [14], evaluated the distribution of SOC with different types of land use [3], [6], the SOC dynamics with vegetation restoration [7], the physical properties of soil under long-term natural vegetation restoration [15] and changes in water storage in soil under landscape restoration [16]. Most of the previous studies were conducted in a small catchment, which may only reflect local SOC dynamics. To the best of our knowledge, few comparable studies on the characteristics of SOC storage and its distribution assessing by soil δ^13^C variation have been conducted in typical restored grassland and secondary forest in the CLP. Therefore, the variation in SOC between typical restored grassland and secondary forest needs to be evaluated to accurately assess the potential differences in soil carbon sequestration.

Variation of SOC between the two vegetation restoration types varies significantly among different studies [17]–[20]. Most studies report a higher SOC content in grassland areas than in forests [7], [21]–[22], while several studies demonstrate little variation between the two, or lower SOC content in the grasslands [14], [23]. Guo and Gifford (2002) evaluated the SOC in forest and grassland, and determined that, on average, grassland accumulates more soil carbon than forest [17]. Nevertheless, the variation of SOC storage and distribution between forest and grassland differs significantly in different regions, and actual measurement of SOC is necessary to explain the variation of SOC among different vegetation types in the CLP.

The variation of soil δ^13^C values with depth, a useful indicator of SOC dynamics [24], is mainly influenced by the SOC decomposition [25], mixing of new carbon with SOC [26] and the Suess effect (the decline of ^13^C atmospheric CO_2_ values with the burning of fossil fuel since Industrial Revolution) [27]. With the topsoil buried, carbon input decreases significantly, and carbon decomposition becomes the main controlling factor of SOC dynamics, which will result in a significant change in soil δ^13^C value. Thus, the measurement of the abundance of natural ^13^C to determine SOC dynamics under different vegetation types has been of increasing interest because of the different ratios of labile and stable carbon and the variance in the SOC source [24].

**Figure 1:**
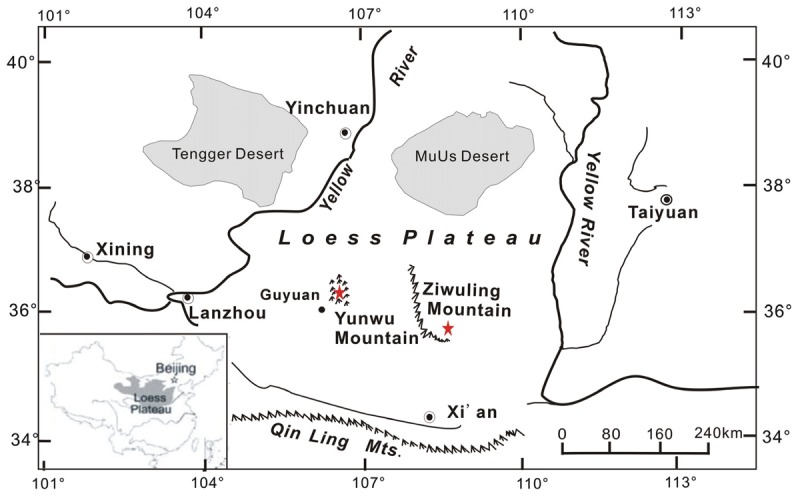
Sample sites (filled red pentagram) of this study at Yunwu Mountain and Ziwuling Mountain.

In an earlier study, we observed a larger SOC content in restored grassland than adjacent farmland at Yunwu Mountain [20]. In this study, we further selected the typical vegetation types in secondary forest and restored grassland (Fig. 1) and determined the SOC content to discuss the effect of different ecotype on soil carbon sequestration. Moreover, we introduced the soil carbon isotopic composition in this study to discuss the different carbon sources and decomposition rate between secondary forest and restored grassland, which cannot be well explained by the data of SOC content. The overall goal of this study was to examine the spatial variability of SOC storage and vertical distribution within sites at typical restored grassland and secondary forest in the CLP. The specific objectives were to 1) quantify the different effects of naturally restored grassland and secondary forest on SOC content and storage in typical vegetation restoration regions in the CLP; and 2) evaluate the characteristics of SOC vertical distribution using soil δ^13^C value and discuss possible mechanisms.

## Results

### Soil bulk density

**Figure 2:**
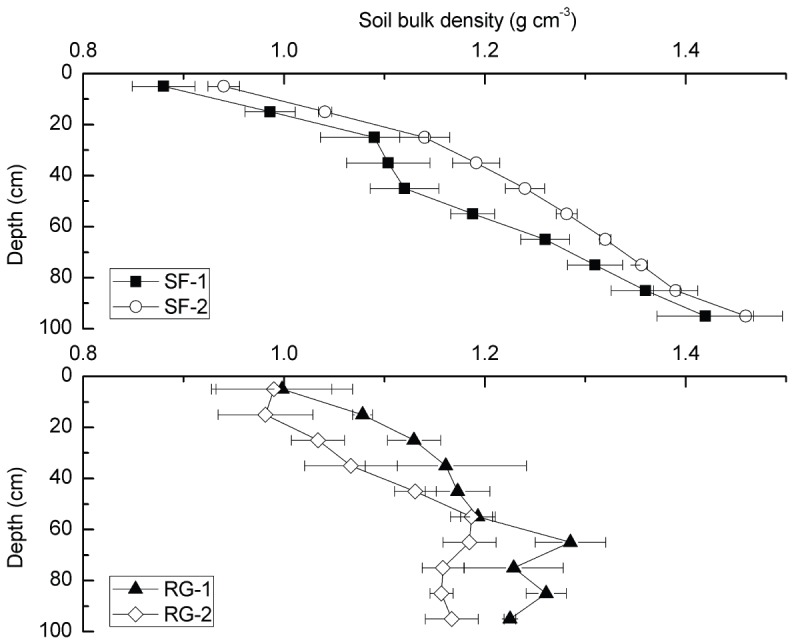
The difference in bulk density at secondary forest (SF-1, SF-2) and restored grassland (RG-1, RG-2). Error bars are standard error (plot to plot and depth to depth, N = 6).

The soil bulk density differed between different types of land use. Fig. 2 illustrated that the bulk density of forestland at SF-1 and SF-2 had a greater range than that of grassland at RG-1 and RG-2. The soil bulk density profile at RG-2 increased up to 1.22 g cm^−3^ and then decreased slightly, whereas the soil bulk density at RG-1 fluctuated at approximately 1.17 g cm^−3^. The bulk density increased gradually throughout both profiles at SF-1 and SF-2 at forest sites.

### SOC content and storage

**Figure 3:**
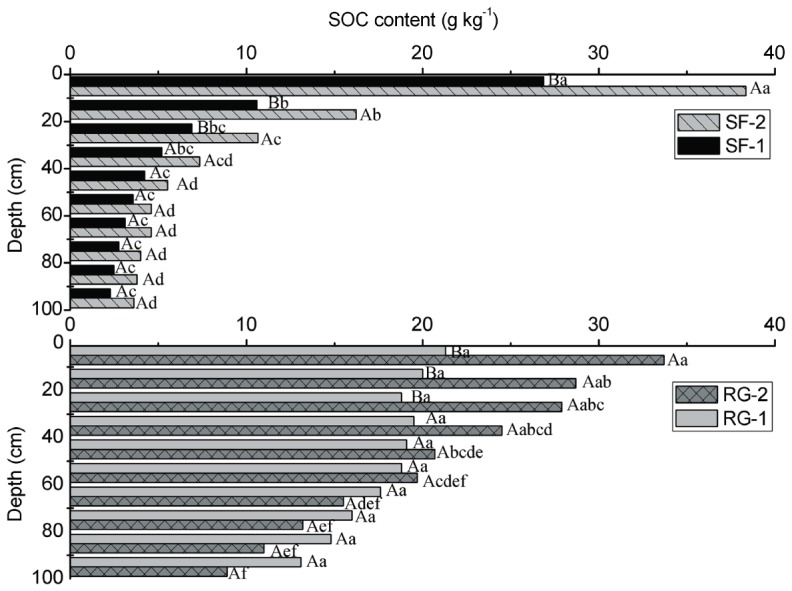
The difference in soil organic carbon (SOC) content. SF-1 and SF-2 are sites of secondary forest while RG-1 and RG-2 are sites of restored grassland. Different lower-case letters denote significant differences among depths within an individual study site; different upper-case letters denote significant differences among vegetation restoration types (*P*<0.05) (plot to plot and depth to depth, N = 6).

**Figure 4:**
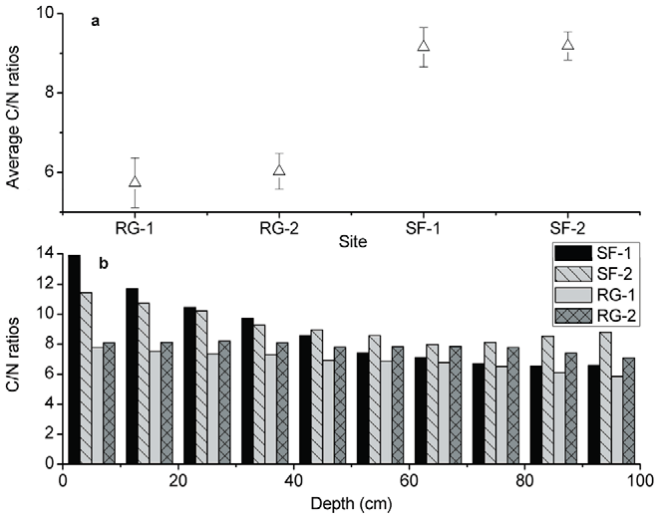
The difference in soil C/N ratios among sites (a) and depths (b). SF-1 and SF-2 are sites of secondary forest while RG-1 and RG-2 are sites of restored grassland. Error bars are standard error (plot to plot and depth to depth, N = 6).

The SOC content decreased with depth at all study sites (Fig. 3). The highest and most variable mean values were in the topsoil at all of the study sites. The average SOC content was 6.8, 9.9, 17.9 and 20.4 g kg^−1^ at sites SF-1, SF-2, RG-1 and RG-2 respectively. The SOC content decreased significantly in the top 40 cm at SF-1 and SF-2 (*P*<0.05) and remained relatively constant at greater depths (Fig. 3). The SOC content did not decrease significantly at the whole depth at RG-1 and in the top 40 cm at RG-2 (*P*>0.05). The SOC contents in each of the soil horizons in grassland at RG-1 and RG-2 were higher than in the forestland at SF-1 and SF-2, except in the top 10 cm. The average C/N ratios of SOC in secondary forest were relatively higher than in restored grassland in the top 100 cm, averaging 8.9, 9.3, 6.9 and 7.8 at SF-1, SF-2, RG-1 and RG-2 respectively. The change of C/N ratios for the whole soil profiles for secondary forest in Ziwuling was significantly more than restored grassland at Yunwu Mountain (Fig. 4).

The average soil carbon storage per unit area was 70.5, 108.6, 210.8 and 223.7 Mg ha^−1^ at SF-1, SF-2, RG-1 and RG-2, respectively, suggesting a significantly higher capacity for SOC sequestration in grassland than in forest areas (Table 1).

**Table 1:** Soil organic carbon (SOC) storage (± standard deviation) at sites secondary forest (SF-1 and SF-2) and restored grassland (RG-1 and RG-2).

	Secondary forest		Restored grassland	
Classes	SF-1	SF-2	RG-1	RG-2
SOC storage per area (Mg ha^−1^)	70.5±19.8	108.6±15.5	210.8±24.6	223.7±34.5
Area (ha)	67600	33400	18	30
SOC storage (10^3^ Mg)	4762.6	3626.7	3.79	6.71
Total SOC storage (10^3^ Mg)	8389.3		10.5	

### Characteristics of soil δ^13^C values

**Figure 5:**
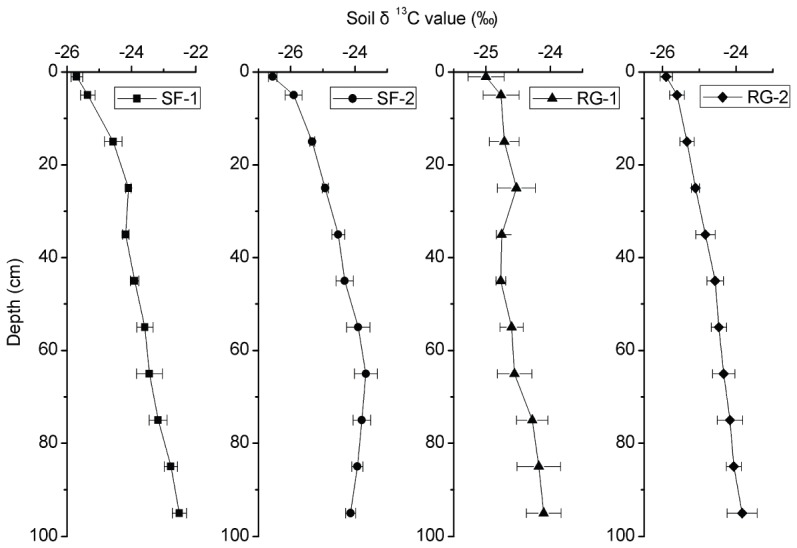
Changes in soil δ^13^C values with depth and vegetation restoration types. SF-1 and SF-2 are sites of secondary forest while RG-1 and RG-2 are sites of restored grassland. Error bars are standard error (plot to plot and depth to depth, N = 6).

Soil δ^13^C value vs. depth curves had similar patterns of variation for the soil profiles of different vegetation restoration sites (Fig. 5). As expected, the most negative values, indicating more input of new carbon, were found in the topsoil. Similar to observed changes in the SOC content, the largest soil δ^13^C changes were found in the surface layers. In forest lands (SF-1 and SF-2), the soil δ^13^C values displayed relatively small variation between the topsoil and depths of 0–10 cm, but showed a significant increase at the top 20 cm, from −25.7‰ and −26.6‰ in the topsoil to −24.6‰ and −25.3‰ at SF-1 and SF-2, respectively, and increased slightly at deeper layers. The soil δ^13^C value showed minor variation with depth at RG-1, with a change of 0.9‰ in the topsoil to depths of 90–100 cm, whereas it increased gradually with depth to a change of 2.1‰ at RG-2 (Fig. 5). The average magnitude of soil δ^13^C was 3.2, 2.9, in SF-1 and SF-2 profiles, respectively.

**Figure 6:**
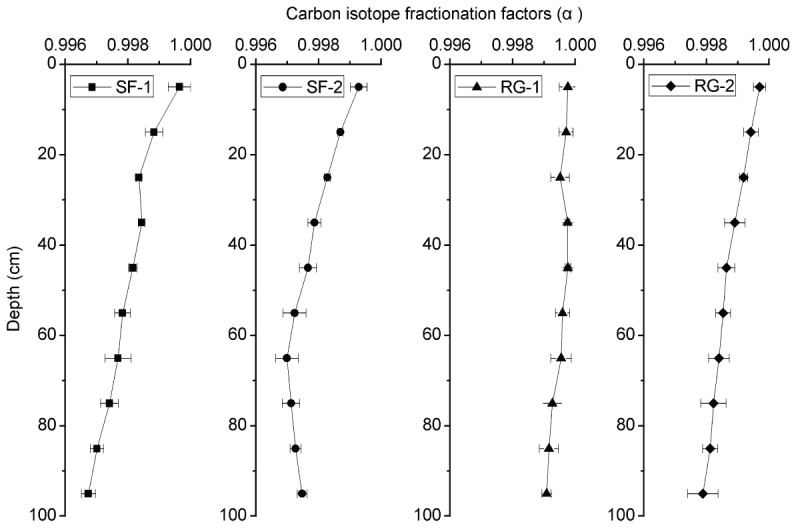
Variation of carbon isotope fractionation factors (α) at different depth among study sites. SF-1 and SF-2 are sites of secondary forest while RG-1 and RG-2 are sites of restored grassland. Error bars are standard error (plot to plot and depth to depth, N = 6).

**Figure 7:**
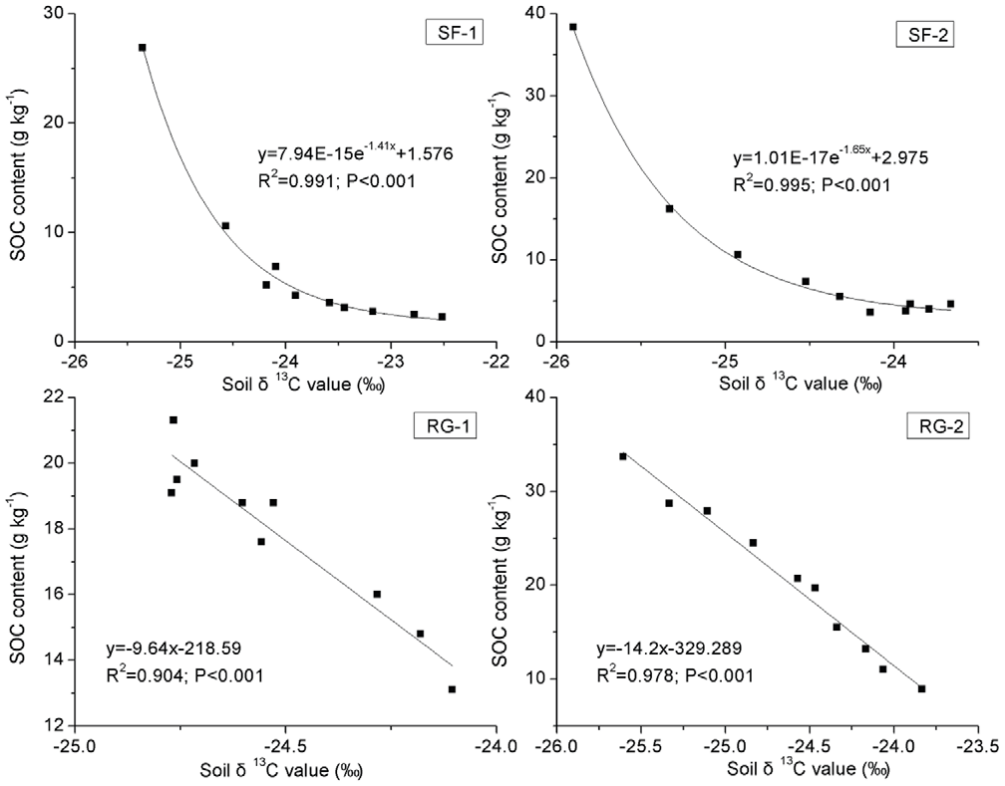
Relationship between soil δ^13^C values and soil organic carbon (SOC) content. SF-1 and SF-2 are sites of secondary forest while RG-1 and RG-2 are sites of restored grassland.

Using equation (3), we calculated the carbon isotope fractionation factors (The quotient of the isotope ratio between two minerals, α_a–b_ = R_a_/R_b_), which were close with each other in the top 10 cm. The carbon isotope fractionation increased with depth both in secondary forest and restored grassland. At a depth of 10–100 cm, the α value was lower in secondary forest than in restored grassland (Fig. 6). Soil δ^13^C vs. SOC content in different vegetation restoration types is depicted in Fig. 7. The soil δ^13^C values increased linearly with the decrease of SOC content in the RG-1 and RG-2 profiles, whereas they increased exponentially with the decrease of SOC content at SF-1 and SF-2.

## Discussion

### Different effect of naturally restored grassland and secondary forest on SOC content and storage

There are many factors and processes that determine the direction and rate of change in SOC content when vegetation and soil management practices are changed [18]. Wang et al. [7] determined the effects of vegetation restoration on SOC sequestration and found different potentials for SOC storage due to different pathways and ecological successions. Dube et al. [28] and Huygens et al. [21] reported an approximately 31% increase and an approximately 42% decrease in SOC content in the conversion from forest to grass and grass to *P. radiata*. Our study at sites SF-1, SF-2, RG-1 and RG-2 is in agreement with the results of previous literature that the restored grassland accumulated significantly more SOC, and thus grass cover is more effective for carbon sequestration.

The mean SOC content was the highest and most variable within the topsoil at all study sites due to large amount of plant litter and root input [29]. The highest SOC contents appeared at SF-2 at forest sites in the topsoil and may be explained by the large amount of aboveground litter fall and relatively low decomposability [18]. The differences in SOC content observed between sites SF-1 and SF-2 at the topsoil were probably due to the different material quality in different forest types and the variance in community vegetation structure [28]. SOC contents decreased sharply in the top 30 cm and nearly approached a constant value at depths of 40–50 cm, demonstrating a slight variation at depths of 50–100 cm. This trend may be attributed to the limited ability of forest to improve SOC content in deep soil. According to Ruiz-Navarro et al. [30], the influence of a 30-year *P. halepensis* reforestation on the soil was minimal, except for the creation of an organic layer due to the low litter quality and dry Mediterranean climate. In New Zealand grasslands, the introduction of pine plantations caused a decrease of SOC and only created a surface litter layer after the occurring of canopy closure [31]. The ecological succession of the grass communities had a significant effect on SOC sequestration [32], whereas no such effect was detected for the forest communities [7]. Therefore, forests may have a limited effect on SOC sequestration.

The SOC content was significantly higher at RG-2 than at RG-1 within the top 30 cm (*P*<0.05) while there was no significant variance at depths of 30–100 cm (Fig. 3). This result may be partly attributed to the differences in distribution of plant roots. According to our field survey, plant roots mainly accumulated within the surface layer at RG-2; in contrast, the roots distributed in deep soil at RG-1. The moderate change of SOC with depth in the top 40 cm in restored grassland, especially at RG-1, may be explained by large root turnover and production and the lower aeration due to the thick root mat reduces the turnover rate of soil organic matter [28].

The higher percentage of SOC content in the top 20 cm at SF-1 and SF-2 than at RG-1 and RG-2 suggested that the distribution of SOC in deeper soils in the grassland than in the forestland is the result of a different source of SOC (Fig. 3). Aboveground inputs in the forestland were the main source of SOC, with tree roots being a less important source because a majority of the trees had grown for many years and there were little root residues. In contrast, in the perennial grassland, the plant roots were the main source of SOC and played a key role in soil carbon sequestration [33]–[34]. Jobbágy and Jackson (2000) also indicated that the decomposition rate of SOC from decaying grass roots is higher than the amount from dying tree roots [35]. The average root to shoot ratios of 3.7 and approximately 0.2 in temperate grasslands and forests, respectively, could partly explain the difference in SOC with depth [36]. The average SOC content in the top 100 cm was 106% higher at RG-2 than at SF-2 (20.4 vs. 9.9 g kg^−1^), where the plant succeeded to natural climax vegetation at Yunwu Mountain and Ziwuling Mountain, illustrating that perennial grass is more effective than woody plants at storing carbon in the study area [17]. That can be attributed to the shorter life cycle of grass and large root residues [28].

The C/N ratios decreased with increased depth in both secondary forest (SF-1 and SF-2) and restored grassland (RG-1 and RG-2), consistent with a build-up of fresh organic matter in shallow layers (with higher C/N ratios) [37] and a depletion of fresh litter in deep layers [32]. An et al. [38] found a greater C/N ratio in the stable water aggregate fractions of the topsoil (0–10 cm) compared with the C/N ratio at depths of 10–20 cm and proposed that raw organic matter was less decomposed in the topsoil. The C/N ratios in secondary forest at Ziwuling were relatively higher than restored grassland at Yunwu Mountain at depths of 0–50 cm, probably due to the natural variation between forest and grass.

### Variance in SOC storage between restored grassland and secondary forest

Arid and semi-arid regions have been regarded as potential carbon sinks recently [39], especially in the CLP, where a large area of farmland was abandoned and converted to forest and grassland [6]–[7]. Vegetation type positively affected the SOC storage in restored fields [19], [32]; therefore, estimating SOC storage in sites covered by different vegetation type is beneficial for accurate assessment of the soil carbon pool.

The average SOC storage was significantly higher at RG-1 and RG-2 than at SF-1 and SF-2, indicating a relatively larger carbon pool in soil in grassland than in forest. This suggests that grassland soil is a more effective carbon sink to mitigate the greenhouse effect in the study area [18], [40]. The average SOC storage at depths of 0–100 cm was higher at SF-2 than SF-1, suggesting that there was a substantial potential for carbon sequestration at SF-1 if the plants succeeded to natural climax vegetation (*Quercus liaotungensis*) in Ziwuling Mountain.

### The characteristics of soil δ^13^C value and its implications for the mechanism of SOC accumulation

Changes of δ^13^C values with depth or decomposition have been widely observed [41]–[48]. In forest lands (SF-1 and SF-2), the soil δ^13^C values exhibited relatively small variation between topsoil and depths of 0–10 cm; however, the values showed a significant increase at the top 20 cm. First, the input of SOM in the forest mainly comes from aboveground leaf litter, with relatively lower δ^13^C values, which mainly accumulates in the topsoil. The mixing of old and new carbon may be the main influencing factor in the lower soil δ^13^C value of topsoil when the soil δ^13^C value is combined with the SOC content [47]. Secondly, the impact of the Suess effect was minimal according to the results at Ziwuling, though it has resulted in a decrease of ^13^C content in the atmosphere by 1.3‰–1.5‰ since the beginning of the Industrial Revolution [27]. The soil δ^13^C values exhibited a slight increase at deeper layers, mainly due to kinetic fractionation during the decomposition of SOM by microbes, resulting in an increased contribution of ^13^C-enriched microbial-derived carbon with depth [43]–[44]. In brief, the sharp increase in the surface layers and slight change in deeper layers of soil δ^13^C value reflects that the main carbon input of secondary forest drives from aboveground litter. The lower soil δ^13^C values at SF-2 than at SF-1 is probably because vegetation restoration has succeeded to the natural climax vegetation and vegetation diversity is greater at SF-2 than at SF-1, reducing the decomposition of SOC by providing inputs with more chemical complexity [49].

There was no sharp increase in soil δ^13^C values throughout the entire profile in the grassland area compared with the forest. This could be attributed to roots presumably being the main source of SOC in the grassland, resulting in large amounts of organic matter input even in deep soil. The mixing of new organic carbon with old carbon resulted in a relatively lower soil δ^13^C value [43], [47]; meanwhile, the percentage of ^13^C-enriched, microbe-derived carbon relatively decreased because of the increase of ^12^C-enriched, root-derived carbon. The gradual increase of the soil δ^13^C values in the top 30 cm, demonstrated that the Suess effect had little influence on the soil δ^13^C value. The soil δ^13^C values were lower at RG-2 than RG-1 in the top 30 cm; however, the values were higher at RG-2 than at RG-1 at depths of 40–100 cm. That can be attributed to the accumulation of new carbon in the surface layers and less new carbon input in deeper layers at RG-2, which ultimately mixed with old carbon and led to a decrease of the soil δ^13^C value. Another reason was the higher average soil water content at RG-2 (12.7%, data not presented) than at RG-1 (10.7%, data not presented), which could result in a decrease in soil δ^13^C value either [45], [50].

Changes of the carbon isotope fractionation factors indicate that the carbon isotopic fractionation increases with depth in all study sites (Fig. 6). According to fractionation factors, there is significantly higher carbon isotopic fractionation in SF-1 and SF-2 than in RG-1 and RG-2 if we only consider carbon decomposition. However, the fractionation factors are undoubtedly influenced by the mixing of new carbon input with old carbon. Thus we believe that the variation of the carbon isotope fractionation factors is the result of new carbon input and carbon decomposition. Compared with the SOC content (Fig. 3), dynamics of the soil δ^13^C values (Fig. 5) with depth are relatively gentler within the surface layer, especially at SF-1 and SF-2 in the forest area [45]. It indicates that although rapid decomposition of organic matter occurs in the surface layer (relative to the deep soil), the ^13^C enrichment remains a slow process, probably because of the higher proportion of new carbon that has a much lower δ^13^C value. On the contrary, even SOC content approached a constant value at depths of 40–100 cm in forest, as ^13^C enrichment continues to take place. This result illustrates that although the resistant organic matter decomposes slowly, it can result in isotopic enrichment [45]. Thus, soil δ^13^C value is a more sensitive index than SOC content in reflecting the changes of SOC. Variations in the soil δ^13^C values with depth correspond well with those of SOC content with depth at our study sites (Fig. 7), indicating that soil δ^13^C value well reflects a comprehensive result of carbon input and decomposition. The soil δ^13^C values increase linearly with the decrease of SOC content at RG-1 and RG-2, but exponentially with the decrease of SOC content at SF-1 and SF-2. That illustrates the different dynamics of SOC between grassland and forest, which are mainly influenced by the input and decomposition rate of SOC, and indirectly by the composition of aboveground vegetation [24].

## Materials and Methods

### Site description and experiment design

We selected four study sites: two sites of secondary forest (SF-1 and SF-2, vegetation restoration for about 50 years) at Ziwuling Mountain and two sites of restored grassland (RG-1 and RG-2, vegetation restoration for about 20 years) at Yunwu Mountain. The dominant species is *Pinus shenkaneusis*, *Quercus liaotungensis*, *Leymus secalinus* and *Stipa grandis* at SF-1, SF-2, RG-1 and RG-2 respectively (Fig. 1). All necessary permits were obtained for the described field studies. We confirm that the field studies do not involve endangered or protected species.

The secondary forest is located in Ziwuling Mountain, which is the sole extensive secondary forest region remaining in the CLP. It covers approximately 23,000 km^2^ and lies at the border of Shaanxi and Gansu provinces, within 35°03′–36°37′N and 108°10′–109°08′E; the elevation ranges between 1,200 to 1,600 m, and the relative relief is approximately 200 m. Air temperature ranges from −27.7°C to 36.7°C, with an annual average temperature of 7.4°C. The average annual relative humidity is between 63% and 68%, and the annual precipitation is 587.6 mm; most of the total annual precipitation falls from June to September. The zonal soil type is calcareous cinnamon soil. The loess thickness is generally low in this region [12]. A large number of inhabitants moved away from the Ziwuling Mountain because of a war between competing tribal groups in 1866; after the farmland was abandoned, large areas were again covered by natural secondary forest.

The restored grassland is located in Yunwu Mountain in the semi-arid area (36°13′–36°19′N and 106°24′–106°28′E) of the CLP in southern Ningxia Province with a total area of 6,000 hm^2^, stretching 4.5 and 11 km from east through west and from north through south. The study region has a sub-arid climate characterized by distinct wet and dry seasons. The annual average temperature is 5°C and precipitation is 423.5 mm (using data from 1980 to 2009). The weather is predominantly controlled by the East Asian monsoon system with about 60% of the annual precipitation falling in the period from July through September. It is the sole typical restored grassland reserve in the CLP [51].

There is little variation in the most recent land use, which were all converted from farmland. The main soil types are Heilu soil and loessal soil in Ziwuling and Yunwu Mountain. All the vegetation consists of C_3_ plants at the study sites.

It is important to note that this is an observational study. The distance between secondary forest and restored grassland is ∼160 km, with somewhat different climates (e.g. precipitation). Many studies have reported that higher rainfall/moisture results in increasing SOC storage [18], [32], [35]. Higher precipitation (587.6 mm) but lower SOC content (6.8 and 9.9 g kg^−1^) in Ziwuling and lower precipitation (423.5 mm) but higher SOC content (17.9 and 20.4 g kg^−1^) in Yunwu mountain suggests that the influence of precipitation on SOC is negligible compared with vegetation types. In the present study, we only chose two representative types of forest in Ziwuling Mountain and two representative types of grass in Yunwu Mountain, but a future study might include more vegetation types.

### Sampling and chemical analysis

To select typical converted secondary forestland and restored grassland, we investigated large areas on the CLP in 2009 and selected the Heshangyuan and Lianjiabian forested farmland areas in Ziwuling, and grassland at Yunwu Mountain as our study sites. Soil samples were collected in June and August 2009 and September 2010. Six soil core samples were taken by a soil borer at 10-cm depth intervals at depths of 0–100 cm at every study site. Meanwhile the topsoil was collected. Then the soil was packed into aluminum foil bags. Meanwhile three replicate samples for soil bulk density analysis (Fig. 2) were taken using a soil corer (stainless steel cylinder of 100 cm^3^ in volume) for each horizon at every study site.

The soil samples for SOC content and isotope composition analysis were dried at 40°C for at least 24 h. Visible roots were removed by hand, ground in an agate mortar, sieved through a 100 mesh screen and homogenized. About 5 g of sieved soil sample was steeped in 2 M HCl for 24 h to exclude the inorganic carbon. The samples were then washed with distilled water until pH>5 and dried at 40°C. The soil samples for bulk density analysis were dried at 105°C for 24 h.

The SOC content was analyzed using an Elemental Analyzer instrument (Vario EL III, Hanau, Germany). Approximately 30 to 50 mg of each soil sample was sealed in a tin can and placed into the automatic sampler to analyze the SOC content. The SOC storage was calculated as follows:
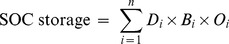
(1)Where *n* is the soil layers, *D_i_* is the soil depth (cm), *B_i_* is the soil bulk density (g cm^−3^), and *O_i_* is the average SOC content (g kg^−1^) at a depth of *i*.

The isotope analysis was dependent on SOC content. Carbon isotope ratios (δ^13^C) were determined using a MAT-252 gas source mass spectrometer with a dual inlet system. Approximately 0.2 to 0.5 g of each soil sample was combusted for 4 h at 850°C in an evaluated sealed quartz tube in the presence of silver foil and cupric oxide and copper. The CO_2_ gas was extracted and purified cryogenically, and the isotope composition of the extracted CO_2_ gas was analyzed with the spectrometer. The ^13^C/^12^C ratio was expressed in δ notation as parts per thousand deviations (‰) from the Pee Dee Belemnite (PDB) standard:
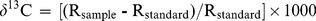
(2)Where R is the ^13^C/^12^C ratio. The analytical precision with running standard (MAT-252) was 0.2‰.

Amundson et al. [52] studied the isotopic fractionation factors (α) and considered that if a soil has had time to reach steady state; the mass balance equations can be greatly simplified. They established the following model of isotopic fractionation factors:
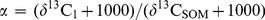
(3)Where α is the isotopic fractionation factors; δ^13^C_I_ and δ^13^C_SOM_ are the carbon isotopic composition of inputs and soil organic matter respectively. The soils in our study sites have been in naturally vegetation restoration for about 20 and 50 years in Yunwu Mountain and Ziwuling Mountain. Thus we consider that they have reached steady state and calculate α value in each study site.

### Statistical analyses

One-way ANOVA followed by the Tukey's HSD test (*P*<0.05) was used to compare the SOC content in different depth intervals in every individual study site. Linear and exponential regression model was fitted to describe the relationships between SOC content and soil δ^13^C value in restored grassland and secondary forest respectively. All statistical analyses were performed using Origin 8 and SPSS 16.0.
